# Supervised Exercise Intervention Restores Physical Activity and MultiOMIC‐Derived Frailty Biomarkers in Older Adults

**DOI:** 10.1002/jcsm.70362

**Published:** 2026-08-09

**Authors:** Diego Marcos‐Perez, Sara Cruces‐Salguero, Morelva Saeteros, Rafael Garcia Molina, Ruben Alcantud‐Córcoles, Cristina Alonso‐Bouzon, Itziar Vergara, Sergio J. Sanabria, Leocadio Rodriguez‐Mañas, Pedro Abizanda, Ander Matheu

**Affiliations:** ^1^ Cellular Oncology Group Biogipuzkoa (Biodonostia) Health Research Institute San Sebastian Spain; ^2^ Deusto Institute of Technology University of Deusto Bilbao Spain; ^3^ Geriatrics Department Complejo Hospitalario Universitario de Albacete Albacete Spain; ^4^ Centro de Investigación Biomédica en Red de Fragilidad y Envejecimiento Saludable (CIBERFES) Instituto de Salud Carlos III Madrid Spain; ^5^ Instituto de Investigación Sanitaria de Castilla‐La Mancha (IDISCAM) Toledo Spain; ^6^ Geriatrics Department Getafe University Hospital Getafe Spain; ^7^ Osakidetza Health Care Directorate PC‐IHO Research Unit of Gipuzkoa Sebastian Spain; ^8^ Primary Care Group Biogipuzkoa (Biodonostia) Health Research Institute San Sebastian Spain; ^9^ Red de Investigacion en Cronicidad, Atencion Primaria y Promocion de la Salud (RICAPPS) Barcelona Spain; ^10^ IKERBASQUE, Basque Foundation for Science Bilbao Spain; ^11^ Department of Radiology Stanford University Palo Alto California USA; ^12^ Facultad de Medicina de Albacete Universidad de Castilla‐La Mancha Albacete Spain

**Keywords:** frailty, OMIC biomarkers, physical exercise intervention, responders

## Abstract

**Background:**

The molecular mechanisms underlying frailty are under intense investigation. Independent observational studies revealed various biological processes and biomarkers differentially expressed in frail individuals and after interventions. Recently, several transcriptomic approaches have described molecular patterns associated with frailty status. However, the effects of physical exercise interventions remain unexplored. Two studies (FRAILOMIC and BIOFRAIL) identified molecular signatures associated with frailty. The objective of the study was to analyse the expression of the FRAILOMIC and BIOFRAIL biomarkers following a multicomponent personalized exercise intervention in older adults.

**Methods:**

A multi‐centre study included a control (*n* = 24, with 14 women and 10 men, median age 80 ± 5) and an exercise intervention group (*n* = 44, with 38 women and 6 men, median age 79 ± 4). The latter underwent a 16‐week multicomponent presential physical exercise programme; clinical, functional and frailty assessments were performed before and after the intervention in both groups. Latent class analysis identified ‘responders’ and ‘non‐responders’ to the intervention. Data in a testosterone‐suppressed control cohort of older men with prostatic cancer receiving androgen deprivation therapy (*n* = 21) were included. Molecular biomarkers were measured via qRT‐PCR and ELISA.

**Results:**

The exercise group presented improvements in functional capacity, as shown by Short Physical Performance Battery (SPPB) (post‐ 10.1 vs. 9.1 in pre‐intervention), grip strength (18.3 vs. 16.9 kg), gait speed (0.85 vs. 0.78 m/s), sarcopenia (2.1 vs. 3.0) and frailty status by Fried Frailty Phenotype (1.6 vs 2.1 criteria) (all ≤ 0.004). ANCOVA revealed significant improvements in SPPB (*p* = 0.04) and grip strength (*p* < 0.005) with exercise. Four of the six FRAILOMIC biomarkers (*miR125b*, *miR194*, *RAGE* and Troponin) and six of seven BIOFRAIL biomarkers (*EGR1*, *CXCL8*, *GOS2*, *NSF*, *DDX11L1* and *miR454*) reversed significantly their expression after exercise training (*p* ≤ 0.05 for FRAIOLMIC and ≤ 0.01 for BIOFRAIL respectively). In contrast, these changes were not observed in testosterone‐suppressed controls, who experienced muscle loss. Latent class analysis revealed 36 ‘responders’ (with 32 cases from intervention group) and 29 ‘non‐responders’. Logistic regression showed that *miR454*, EGR1, *DDX11L1* and Troponin displayed the strongest predictive value in distinguishing responders from non‐responders.

**Conclusions:**

Exercise intervention improved functional status and reversed biological frailty, as evidenced by changes in multiple MultiOMIC frailty biomarkers. Among these, the BIOFRAIL trio—*miR454*, *EGR1* and *DDX11L1*—showed the strongest predictive power for intervention response with AUC of 0.83. This study provides proof‐of‐concept for the utility of OMIC‐derived frailty biomarkers as outcome measures after intervention.

## 1 Introduction

The World Health Organization defines frailty as a clinically recognizable state in which the ability of older people to cope with everyday or acute stressors is compromised due to age‐associated declines in physiological reserve and function across multiple organ systems [1]. Frailty precedes dependency and is linked to falls, diminished quality of life, hospitalization, institutionalization and increased mortality [2]. It also significantly increases healthcare resource utilization and costs [3] and is recognized globally as an emerging public health challenge [4, 5].

Frailty detection has historically been challenging in clinical settings, leading to the development of various functional tests, questionnaires and indices. Currently, two major constructs—the Frailty Phenotype [6] and the Frailty Index [7]—are well validated and widely used among clinicians and researchers. To supplement these clinical tools, extensive studies have aimed to identify biological markers of frailty. Most knowledge arises from observational studies, which indicate that high oxidative stress, chronic low‐grade inflammation, immune dysregulation and epigenetic changes are the biological processes most frequently associated with frailty [8, 9, 10]. Nevertheless, clinical frailty biomarkers have shown inconsistent replication across cohorts. Recently, MultiOMIC studies have explored the molecular pathways underlying frailty. The FRAILOMIC Consortium [11, 12] was the first to employ a MultiOMIC approach in eight European cohorts totalling over 75 000 individuals, uncovering nine frailty‐related biomarkers. Among these, three miRNAs (*miR125* and *miR454* lower and *miR194* higher in frail individuals) and four proteins (Troponin‐T, pro‐BNP and RAGE increased; Vitamin D decreased) were significantly associated with frailty status [12]. In parallel, we reported the BIOFRAIL transcriptomic study, identifying a seven‐gene pattern linked with frailty, notably increased *G0S2*, *EGR1*, *CXCL8* and *GJB6* and lower *NSF*, *DDX11L1* and *miR454* expression validated across three cohorts. The minimum gene trio—*EGR1*, *DDX11L1* and *miR454*—showed strong potential for identifying frail individuals.

Frailty is a dynamic and potentially reversible condition, improved by interventions such as exercise [13], nutrition [14], inappropriate medication control [15] or multicomponent approaches [15]. Of these, physical activity is most established for counteracting frailty and age‐related diseases [16], especially when individually tailored as multicomponent programmes, and is recommended for healthy longevity and frailty prevention [17, 18, 19].

Studying the molecular response to exercise is crucial for validating frailty biomarkers [20, 21]. Past studies show that physical interventions in frail individuals reduce inflammation [22, 23], senescence biomarkers [24, 25] and improve mitochondrial function, myokine profile, insulin‐like growth factor‐1 (IGF‐1) signalling and insulin sensitivity [26]. This study aimed to assess changes in MultiOMIC‐derived frailty biomarkers (FRAILOMIC and BIOFRAIL sets) following a physical intervention in older adults.

## 2 Material and Methods

### 2.1 Study Setting and Population

Patients were recruited consecutively by the Geriatrics Departments of the University Hospital of Getafe (control) and the Complejo Hospitalario Universitario de Albacete (exercise group) at the outpatients clinics. Inclusion criteria: Women and men with an age equal to or over 70 years old, ability to walk, ability to follow a physical exercise programme, ability to perform all the functional tests and provide signed informed consent. Exclusion criteria: Expected survival inferior to 1 year, Barthel index < 70 (moderate dependency in basic activities of daily living), moderate to severe cognitive impairment, refusal to participate, medical conditions that may condition or difficult the follow‐up assessments as assessed by the investigators and older adults already included in regular physical exercise programmes. Recruitment was conducted between February 2022 and March 2023. For the study objective, we estimated to recruit a sample size of 100 participants that provides 80% power to detect a biomarker with a medium effect size (E/S = 0.284) at a two‐sided α level of 0.05. The sample size was calculated using RiskCalc (Moody's Analytics) [27]. The cohort included 98 participants (32 controls and 66 exercisers) with similar rates of adherence: control (75%) and exercise (66.7%), resulting in a final sample of 24 controls and 44 exercisers (Figure S1). Adherence was measured with the percentage of completed sessions. Dropouts were mainly due to voluntary physical exercise programme abandonment. There were neither deaths nor adverse events coming from the exercise programme. Controls received usual care for 16 weeks. The exercise group underwent a supervised, tailored, 16‐week multicomponent physical programme. The 16‐week multicomponent exercise intervention was individualized based on the functional capacity, comorbidities and previous experience of the subject. On a weekly basis, subjects performed two supervised sessions of 45 min in small groups (4‐to‐6 older adults). The sessions were divided into warm‐up, main part (strength, power and coordination exercises) and cool down (flexibility and stretching). The main part contained eight exercises that worked the main muscle groups, starting with low intensity and high‐volume sessions and progressing to higher intensities and lower volumes. Depending on the stage, patients performed two to four sets, 4–15 repetitions with 30‐s rest between sets and exercises. For every exercise, loads and intensities were modulated to allow a total of 30 repeats. The exercise sessions were to be performed in the gym of the Geriatrics Department of the hospital. In addition, participants received recommendations to take additional 90‐min walks per week. It should be noted that the programme was based on the guidelines of the VIVIFRAIL programme [28, 29] aimed at preventing frailty, weakness and the risk of falls. After the follow‐up period or the completion of the exercise programme, a repetition of the baseline clinical and blood‐based evaluation was conducted.

Samples from PRO‐SARC study were also analysed. PROSARC is an observational study carried out in the hospitals of the Albacete University Hospital Complex. It included patients aged ≥ 70 years, high‐risk of prostate cancer, not candidates for local treatment, and performing Androgen deprivation therapy (ADT) according to clinical practice. Detailed information can be obtained in Protocol study [30].

### 2.2 Clinical Measurements

Briefly, clinical evaluation protocol assessed frailty by three scales, FRAIL (short five‐question assessment of Fatigue, Resistance, Aerobic capacity, Illnesses and Loss of weight) scale ranging from 0 to 5, with 0 indicating robustness and 5 indicating frailty, by Frailty phenotype, ranging from 0 (robust) to 5 (frail), and by the Short Physical Performance Battery (SPPB) test ranging from robust (10–12 points), pre‐frail (7–9 points) and frail (4–6 points) as previously described in the Vivifrail study. SPPB test also was used to measure physical function, ranging from 0 to 12, where 0 indicates the lowest physical performance and 12 indicates the highest performance. Sarcopenia was assessed by SARC‐F (Strength, Assistance with walking, Rising from a chair, Climbing stairs, and Falls) scale ranging from 0 (robust) to 10 (sarcopenic), International Physical Activity Questionnaire scores ranging from 0 (lowest) to 3 (highest), Gait Speed Test in seconds, grip strength measured by Jamar dynamometer in kilogram, disability (Global Deterioration Scale, consisting of seven stages), Barthel Index (ranging from 0 [total dependent] to 100 [independent]), Lawton and Brody Index (scores from 0 [dependent] to 8 [independent]), nutritional status (Mini Nutritional Assessment—Short Form) and quality of life (EQ‐5D‐5L).

### 2.3 Ethical Considerations

The study adhered to the Declaration of Helsinki, with approvals by the Research Ethics Committee of Albacete reference (CEIm‐2021‐51) and the Clinical Research Ethics Committee of Euskadi (PI2022069). Before participation, written informed consent was obtained from all participants.

### 2.4 Enzyme‐Linked Immunosorbent Assay (ELISA)

Plasma protein levels were measured using commercial enzyme‐linked immunosorbent assay (ELISA) kits, including Human EGR1 ELISA Kit (EH167RB, Invitrogen), Human RAGE Quantikine ELISA Kit (DRG00, R&D Systems), Human Cardiac Troponin T ELISA Kit (ab223860, Abcam), Human pro‐BNP ELISA Kit (ab193712, Abcam) and Human 25(OH) Vitamin D ELISA kit (ab213966, Abcam).

### 2.5 Quantitative Real‐Time Polymerase Chain Reaction (qRT‐PCR)

RNA samples from serum were extracted using the Maxwell RSC miRNA Plasma and Serum Kit (AS1680, Promega). RNA concentration was determined with a Nanodrop‐100 spectrophotometer (Thermo Fisher). Reverse transcription (RT) was performed using the Maxima First Strand cDNA Synthesis Kit (K1671, Thermo Fisher). A 10–20 ng of cDNA was used as the reaction template with Power SYBR Green Master Mix (4368706, Applied Biosystem). For the study of miRNAs, the miRCURY LNA SYBR Green PCR kit (339347, bioNova) was employed. Primers were designed to span exon‐exon junctions in order to avoid amplification of genomic DNA. As internal controls, the housekeeping genes glyceraldehyde 3‐phosphate dehydrogenase (*GAPDH*) and beta‐2‐microglobulin (*B2M*) were used. For miRNA normalization, *miR191‐5p* served as the housekeeping reference. Relative quantification was calculated using the 2^−ΔΔCt^ method.

### 2.6 Statistical Analysis

Descriptive statistics were applied to both Control and Intervention cohorts independently, in order to analyse clinical data before and after the intervention period. Mean and standard deviation (SD) were reported for both continuous and ordinal variables, while counts and frequencies (%) were calculated for categorical variables. Difference in means between pre‐ and post‐intervention measures were calculated. Shapiro–Wilk test was employed to assess the normality of numerical variables, and a paired Student's *t*‐test or Wilcoxon signed‐rank test was used depending on the distribution of the data.

Gene expression data from qRT‐PCR were processed using the −ΔΔCt method, with pre‐intervention measures as the reference. Outliers were identified and removed based on Principal Component Analysis (PCA) and expression heat maps, leaving a total of 21 samples in the Control group and 44 in the Intervention group. Differences in gene and protein expression before and after the intervention were assessed in each cohort independently using a paired *t*‐test or Wilcoxon signed‐rank test, as appropriate. The results were presented as fold‐change relative to pre‐intervention measures. The intervention effect was assessed using ANCOVA for both expression and clinical variables.

For the following multivariate analyses, gene and protein expression ratios and clinical variable differences between pre‐ and post‐intervention were employed. Latent class analysis (LCA) was used to search for common patterns of physical response among both Control and Intervention cohorts. Cohort group, gender, SPPB change, frailty phenotype change and sarcopenia change from pre‐ to post‐intervention were considered, and a model with two latent classes was finally built. Descriptive statistics were applied to compare the resultant classes, which were defined as ‘responders’ and ‘non‐responders’. Mann–Whitney *U* test and chi‐squared test were used for ordinal and categorical variables, respectively.

The relation between class membership and gene and protein expression ratios was assessed through a support vector machine (SVM) model. To address missing values, multiple imputation by chained equations was applied prior to model building. The performance of the SVM in classifying responders and non‐responders was assessed through a confusion matrix, and accuracy, sensitivity, specificity and precision were calculated. The weight of each feature in the model was calculated to identify the most relevant variables for the classification. The three genes and protein ratios with the highest weights were included in a logistic regression model to distinguish between responders and non‐responders, built with a training set (70% of the total sample) and validated on the test set (remaining 30%). Odds ratio (OR) with a 95% confidence interval (CI) was used as the measure of association. Calibration was measured by the Hosmer‐Lemeshow test. The predictive power of the models and individual biomarkers was assessed through ROC curves.

The statistical analysis was conducted using R in RStudio software (version 4.3.1, URL: https://www.R‐project.org/). Univariate analyses graphs were generated using GraphPad Prism version 9.4.1 (Boston, MA, USA). Statistical significance was established at *p* < 0.001 (***); *p* < 0.01 (**); *p* < 0.05 (*); and *p* < 0.1 (≠).

## 3 Results

**FIGURE 1:**
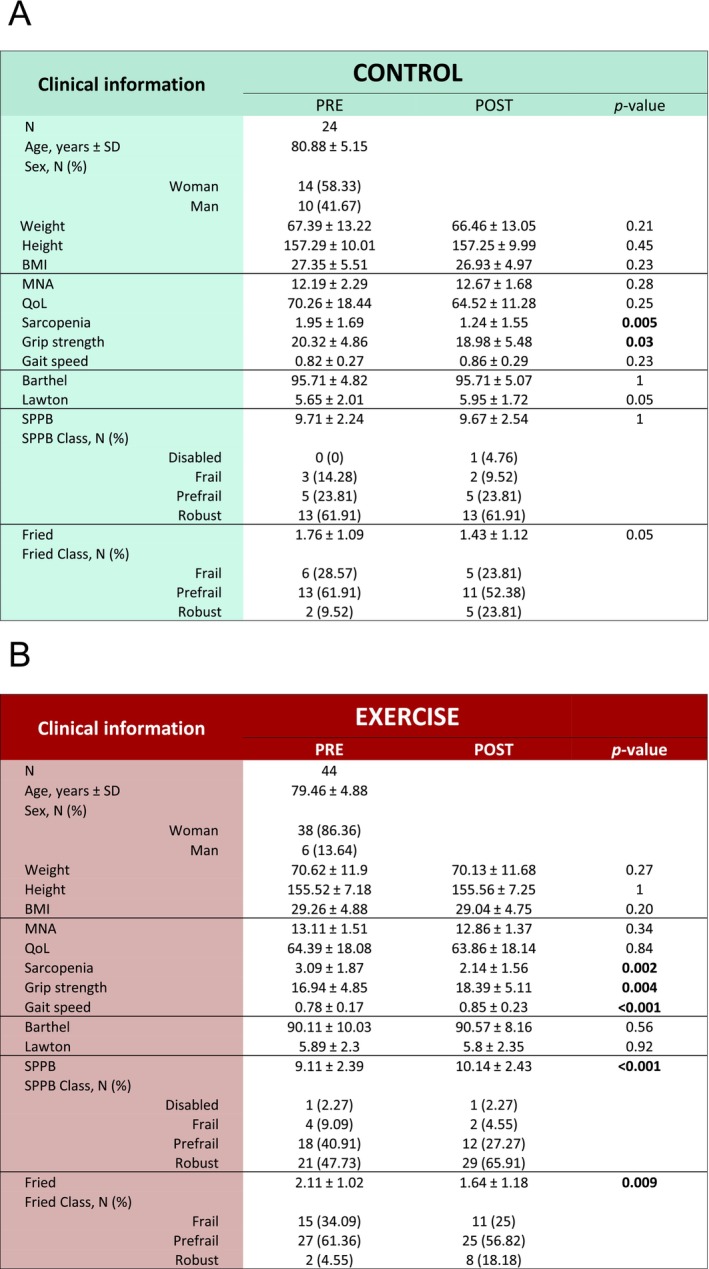
Clinical characteristics of participants. (A, B) Main clinical characteristics of participants in control group (green) and in exercise intervention (red).

Control and exercise groups were characterized at baseline, and they showed similar clinical characteristics (age, BMI, etc.), functional measurements (Barthel, Lawton, gait speed, grip strength), frailty phenotype and physical function (Short Physical Performance Battery [SPPB]) (Table 1). The exercise group had a higher proportion of women compared to the control group. Participants did not differ between groups in comorbidity or commonly used drugs as described in Table 1. Moreover, participants did not change their medications or their dietary habits between baseline and follow‐up visits. Nutritional status measured with MNA‐SF scale was within normal ranges in both cohorts, although significant differences were observed between the control and exercise groups, with the latter showing higher scores (11.97 vs. 13.06) (Table 1). These results indicated that there were no baseline differences between the groups for most clinical and functional variables. Similarly, PCA analysis showed no differences across all variables in both cohorts or in gene expression under pre‐intervention conditions (Figure S2A,B). However, the intervention resulted in significant differences in both groups. The control group showed a significant decline in grip strength (Figure 1A), whereas the 16‐week multicomponent physical exercise group experienced significant improvements in grip strength, gait speed and SPPB (Figure 1B). Besides, frailty, as measured by the frailty phenotype, improved significantly after the 16‐week physical training (Figure 1B). Next, we compared the functional direct effects of the physical exercise intervention in both groups performing an ANCOVA analysis. Notably, the exercise intervention led to improvements in several measures, including grip strength, SPPB, MNA‐SF and BMI (Table 2). These results revealed the benefits of the 16‐week personalized exercise training on the clinical and functional status of older individuals.

**TABLE 1:** Sociodemographic and clinical characteristics of study participants at baseline.

Baseline measurements	Control	Exercise	*p*
*N*	32	66	
Age, years ± SD	81.02 ± 5.04	80.08 ± 5.34	0.35^b^
Sex, *N* (%)			
Woman	18 (56.25)	56 (84.85)	
Man	14 (44.75)	10 (15.15)	
Weight	69.11 ± 14.58	69.96 ± 12.91	0.45^b^
Height	157.90 ± 9.39	156.0 ± 7.42	0.40^b^
BMI	27.76 ± 5.58	28.78 ± 5.10	0.21^b^
MNA	11.97 ± 2.31	13.06 ± 1.50	**0.01** ^**b**^
QoL	70.33 ± 17.95	66.38 ± 16.19	0.25^b^
Sarcopenia	2.47 ± 1.83	2.97 ± 1.76	0.17^b^
Grip strength	18.77 ± 5.55	17.14 ± 5.38	0.16^a^
Gait speed	0.82 ± 0.25	0.76 ± 0.20	0.20^a^
Barthel	93.28 ± 7.47	90.91 ± 9.76	0.23^b^
Lawton	5.55 ± 1.91	6.03 ± 2.15	0.11^b^
SPPB	9.59 ± 2.15	8.88 ± 2.63	0.22^b^
Disabled	0 (0%)	3 (4.54%)	
Frail	4 (12.50%)	7 (10.60%)	
Prefrail	8 (25.00%)	24 (36.36%)	
Robust	20 (62.50%)	32 (48.48%)	
Fried	1.97 ± 1.06	2.15 ± 1.18	0.45^b^
Frail	2 (6.25%)	5 (7.58%)	
Prefrail	22 (68.75%)	36 (54.54%)	
Robust	8 (25.00%)	25 (38.38%)	
Charlson index	1.40 ± 0.25	1.62 ± 0.20	NS
Hypertension	24 (77.4%)	51 (77.3%)	NS
Type 2 diabetes	10 (31.3%)	23 (34.8%)	NS
Heart disease	1 (3.1%)	3 (4.5%)	NS
Dementia	2 (6.3%)	6 (9.1%)	NS
Chronic Kidney disease	3 (9.4%)	5 (7.6%)	NS
Number of common used drugs	6.1 ± 3.0	6.4 ± 2.5	NS

*Note:* Analysis of baseline variables in whole control (*n* = 32) and intervention (*n* = 66) groups. Bold numbers represent statistical significance (*p* < 0.05).

Abbreviations: BMI: body mass index; MNA: Mini Nutritional Assessment; QoL: quality of life; SPPB: Short Physical Performance Battery.

^a^ Unpaired T test.

^b^ Mann–Whitney test.

**TABLE 2:** Univariate and ANCOVA control (*n* = 22) and intervention (*n* = 44) clinical results before and after 16‐week period.

Clinical variables	Control				Intervention				ANCOVA
	Pre	Post	Δ_Post‐Pre_	*p*	Pre	Post	Δ_Post‐Pre_	*p*	*p*
SPPB	9.7 ± 2.2	9.6 ± 2.5	−0.05 ± 1.7	1^b^	9.1 ± 2.3	10.1 ± 2.4	1 ± 1.8	**< 0.001** ^b^	**0.04**
Fried	1.7 ± 1.0	1.4 ± 1.1	−0.3 ± 0.7	0.05^b^	2.1 ± 1.0	1.6 ± 1.1	−0.5 ± 1.2	**0.009** ^b^	0.97
Sarcopenia	1.9 ± 1.6	1.2 ± 1.5	−0.7 ± 1	**0.005** ^b^	3.0 ± 1.8	2.1 ± 1.5	−1 ± 1.8	**0.002** ^b^	0.15
Grip strength	20.3 ± 4.8	18.9 ± 5.4	−1.4 ± 2.7	**0.03** ^a^	16.9 ± 4.8	18.3 ± 5.1	1.6 ± 3.3	**0.004** ^a^	**0.006**
MNA	12.1 ± 2.2	12.6 ± 1.6	0.5 ± 2.4	0.28^a^	13.1 ± 1.5	12.8 ± 1.3	−0.25 ± 2	0.34^a^	**0.01**
BMI	27.3 ± 5.5	26.9 ± 4.9	−0.4 ± 1.3	0.23^b^	29.2 ± 4.8	29.0 ± 4.7	−0.1 ± 0.7	0.20^b^	*0.05*
QoL	70.2 ± 18.4	64.5 ± 11.2	−6 ± 20.7	0.25^b^	64.3 ± 18.0	63.8 ± 18.1	−0.5 ± 24.6	0.84^b^	0.98
Gait speed	0.82 ± 0.2	0.86 ± 0.2	0.04 ± 0.2	0.23^a^	0.78 ± 0.1	0.85 ± 0.2	0.1 ± 0.1	**< 0.001** ^b^	0.22
Barthel	95.7 ± 4.8	95.7 ± 5.0	0 ± 4.7	1^a^	90.1 ± 10.0	90.5 ± 8.1	0.5 ± 5.2	0.56^a^	0.40
Lawton	5.6 ± 2.0	5.9 ± 1.7	0.4 ± 1.1	0.05^b^	5.8 ± 2.3	5.8 ± 2.3	−0.1 ± 1.1	0.92^b^	0.82

*Note:* Bold numbers represent statistical significance (*p* < 0.05).

Abbreviations: BMI: Body Mass Index; MNA: Mini Nutritional Assessment; QoL: Quality of Life; SPPB: Short Physical Performance Battery.

^a^ Paired T test.

^b^ Wilcoxon test.

**FIGURE 2:**
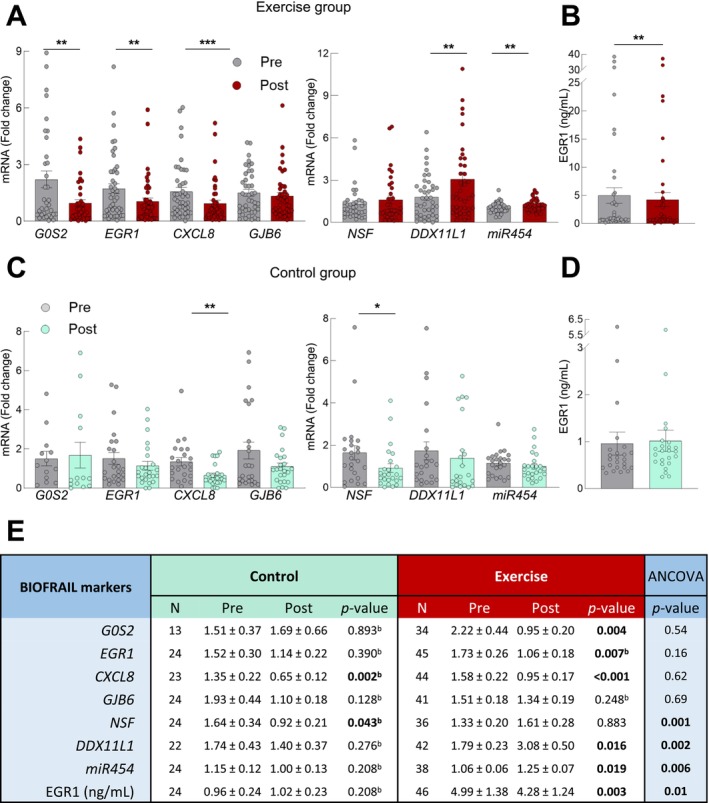
Multicomponent physical exercise intervention reverses BIOFRAIL study derived frailty molecular biomarkers. (A) mRNA levels of the seven candidate genes (stratified by high and low expression in frail individuals) in samples from before and after the 16 weeks of multicomponent exercise intervention group. (B) EGR1 protein levels in samples from before and after 16 weeks of multicomponent exercise intervention group. (C) mRNA levels of the seven candidate genes in samples from control group. (D) EGR1 protein levels in samples from control group. (E) Univariate and ANCOVA analyses of the seven candidate genes. Data as mean ± standard error of mean (SEM). Sample sizes (*N*) for individual biomarkers may vary from the total clinical follow‐up sample (*n* = 24 for controls; *n* = 44 for the exercise group) due to technical laboratory limitations, including insufficient serum volume or qPCR amplification failures. All available data were included in the analysis.

Next, we characterized the molecular expression profiles of the BIOFRAIL and FRAILOMIC studies. First, we studied the expression of the seven genes from BIOFRAIL by qRT‐PCR. Notably, the exercise group showed a statistically significant reduction in *G0S2*, *EGR1* and *CXCL8* expression, along with a significant increase of *DDX11L1* and *miR454* expression in post‐intervention samples (Figure 2A). EGR1 protein levels by ELISA were also reduced in post samples (Figure 2B). In contrast, no reversion or significant differences were observed in mRNA or protein expression in the control group, except for a significant decline in *CXCL8* levels (Figure 2C,D). To assess the effect of the exercise programme on the selected genes, ANCOVA was performed, revealing significant improvements in *NSF*, *DDX11L1* and *miR454‐3p* expression (Figure 2E). These data confirm that the expression of several biomarkers, including the trio composed of EGR1, DDX11L1 and miR454 from the BIOFRAIL study, is modulated by physical intervention training in older adults.

**FIGURE 3:**
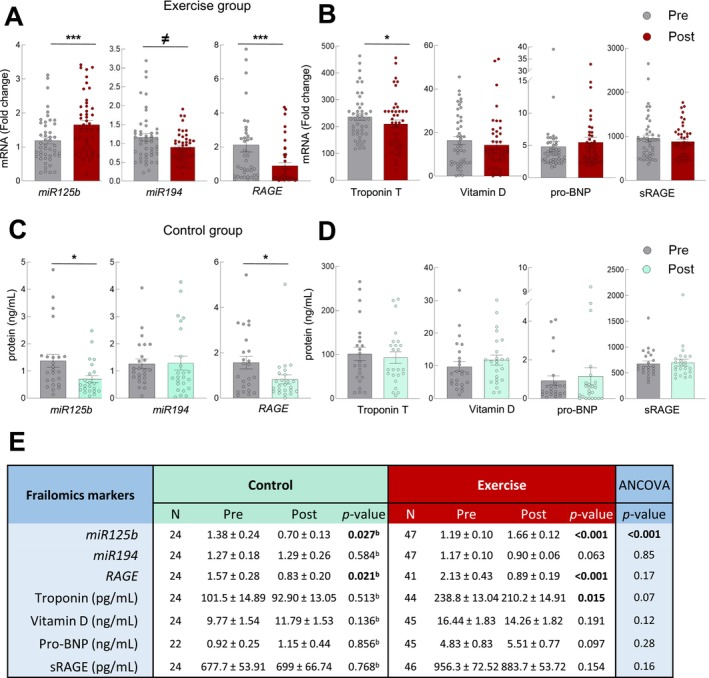
Multicomponent physical exercise intervention reverses FRAILOMIC study derived frailty molecular biomarkers. (A and B) mRNA levels of three genes and protein levels of four in samples from before and after the 16 weeks of multicomponent exercise intervention group. (C and D) mRNA levels of three genes and protein levels of four in samples from control group. (E) Univariate and ANCOVA analyses of the seven candidate genes. Data as mean ± standard error of mean (SEM). Sample sizes (*N*) for individual biomarkers may vary from the total clinical follow‐up sample (*n* = 24 for controls; *n* = 44 for the exercise group) due to technical laboratory limitations, including insufficient serum volume or qPCR amplification failures. All available data were included in the analysis.

To further investigate the molecular characterization, we checked the biomarkers reported by FRAILOMICS consortium [12]. For this purpose, we determined the levels of *miR125b*, *miR194* and *RAGE* levels by qRT‐PCR, as well as the protein levels of Troponin T, Vitamin D, pro‐BNP and sRAGE. Remarkably, we found a significant reversal in *miR125b*, *miR194* and *RAGE* mRNA levels, as well as Troponin T protein, in the intervention group (Figure 3A,B). In contrast, only *RAGE* expression was restored in the control group (Figure 3C,D). ANCOVA confirmed significant improvements in *miR125b* and Troponin T expression as a result of the exercise training programme (Figure 3E). These results show the reversal of several FRAILOMIC biomarkers following physical intervention in older adults.

**FIGURE 4:**
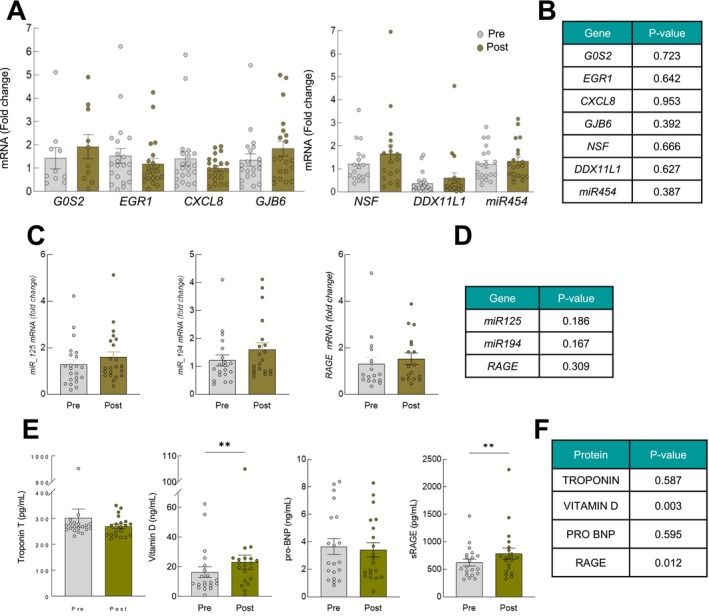
No differences in BIOFRAIL and FRAIOLMIC biomarkers in androgen deprivation therapy. (A) mRNA levels of three genes and protein levels of four in samples from ADT group. (B) Univariate analyses of the seven candidate genes. (C and D) mRNA levels of three genes and Univariate analyses. (E and F) Protein levels of four indicated genes and univariate analyses.

Androgen deprivation therapy (ADT) is a common therapy for high‐risk prostate cancer patients [31], producing testosterone suppression and inducing metabolic changes such as increased weight and fat mass, along with decreased muscle mass [32]. To investigate whether the improvements in frailty biomarkers expression are due to exercise training or secondary to muscle alteration, we determined their levels on a set of patients of prostate cancer treated with ADT, which might serve as a specificity control, since this cohort is age‐matched with the primary study population, although present oncological disease and a distinct endocrine‐driven muscle loss trajectory compared to age‐related sarcopenia. First, we characterized the cohort in terms of demographic data and cancer information (Figure S3A,B) and found that ADT significantly improved the cancer severity based on PSA and testosterone levels (Figure S3C), without affecting**—**or at least not improving—the functional and frailty status (Figure S3D). In this context, no significant differences were observed in any of the 7 BIOFRAII genes between pre‐ and post‐therapy samples (Figure 4A,B). For the FRAILOMIC biomarkers, only Vitamin D and RAGE protein levels were altered by the therapy (Figure 4C–F), with changes in Vitamin D occurring in the same direction as observed with the exercise intervention. The lack of alterations in the majority of BIOFRAIL and FRAILOMIC biomarkers during ADT suggests that these molecules are indicators of frailty status and exercise‐induced reversal, rather than general bystanders of acute muscle mass depletion.

**FIGURE 5:**
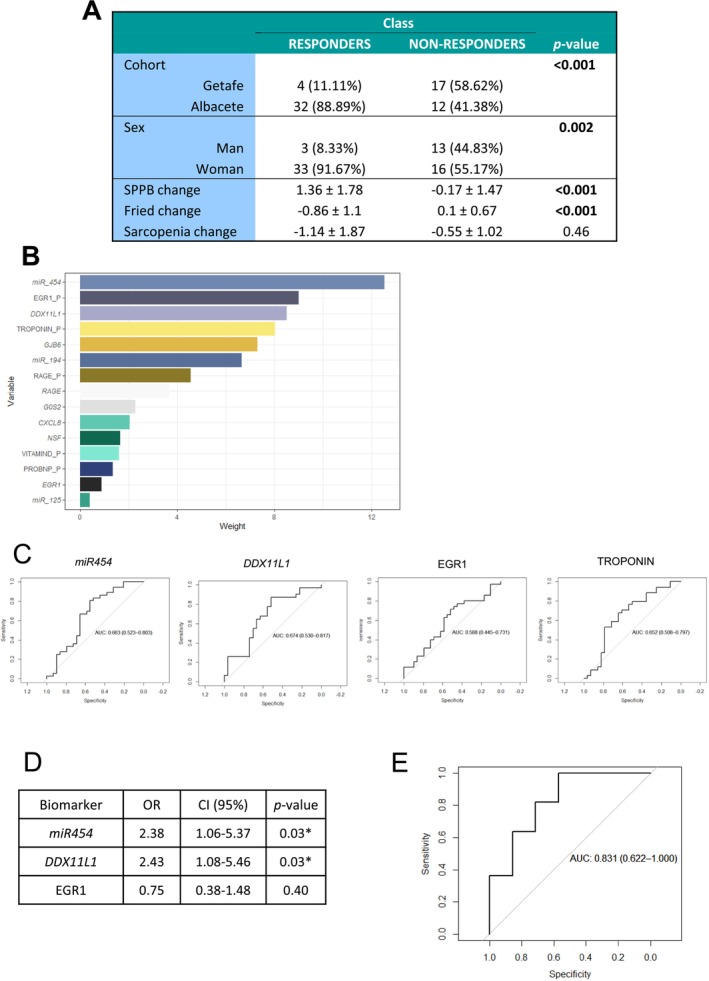
Generation of ‘responders’ by latent class analyses. (A) ‘Responders’ and ‘non‐responders’ cluster clinical description. (B) Weight of each biomarker in the SVM model classification. (C) ROC curves of the four most important biomarkers in the SVM model. (D) Logistic regression model of the three most important biomarkers. (E) ROC curve of the logistic regression model.

Taking into account the inter‐individual heterogeneity observed in the number of sessions and the responses of some participants in both groups—where a few individuals improved their functional status in the control group while others worsened in the intervention group, and greater changes in the frailty phenotype were detected with increasing session numbers (Figure S4A–D), we performed a latent class analysis to identify clusters of functional improvement in the entire ECOFRAIL cohort. First, we combined clinical data from both cohorts and considered three frailty‐related factors: SPPB, frailty phenotype and sarcopenia changes, along with cohort and gender. LCA divided the whole sample into two different classes, which we termed ‘responders’ and ‘non‐responders’ (Figure S5). In brief, the ‘responders’ class mainly comprised women from the exercise group who showed significant improvements in both SPPB and frailty phenotype (Figure 5A). Contrarily, the ‘non‐responders’ class included a more balanced representation of participants from both the control and exercise groups, with nearly equal gender distribution, and showed either no change or worsening in SPPB and frailty phenotype (Figure 5A). To assess whether the selected biomarkers could distinguish between ‘responders’ and ‘non‐responders’, we built a SVM model using gene and protein ratios as predictors and the two latent classes as the outcome. The model demonstrated strong classification performance, with accuracy (92.31%), sensitivity (97.22%), specificity (86.21%) and precision (89.74%). The weight of each variable was then calculated to identify the biomarkers contributing most to this classification (Figure 5B). The three most relevant biomarkers were *miR454*, *DDX11L1* and EGR1, which showed independent AUCs of 0.663, 0.674 and 0.588, respectively (Figure 5C). The fourth biomarker, and the first one from the FRAILOMIC study, was Troponin T, with an AUC of 0.652 (Figure 5B,C). These results are consistent with the ANCOVA intervention effect results described in Figures 2G and 3E. Finally, the three biomarkers (*miR454*, *DDX11L1* and EGR1) were used to build a logistic regression model, which revealed that each unit increase in the expression ratios of *DDX11L1* and *miR454* was associated with increased odds of being a ‘responder’ after a physical intervention by a factor of 2.38 and 2.43, respectively (Figure 5D). Those associations remained, particularly in the case of *miR454*, even after adjusting for sex, age and SPPB values at baseline (Figure S6A). Calibration measured by Hosmer‐Lemeshow test showed no significant differences between observed and predicted values (*p* = 0.051, Figure S6B). Besides, the ROC curve derived from the model suggested a predictive potential (AUC = 0.831 [0.622–1.000]) for classifying responders and non‐responders, despite the limited sample size.

## 4 Discussion

The definition of a biomarker is ‘a characteristic that is objectively measured and evaluated as an indicator of normal biological processes, pathogenic processes, or pharmacologic responses to a therapeutic intervention’ [33]. In the context of frailty, a biomarker would facilitate the identification of individuals who are frail, enable greater tracking of frailty over time, help optimize treatments and interventions and allow ease monitoring of whether interventions are working [8]. There are currently no accepted biomarkers of frailty, a challenge that researchers are trying to overcome through unbiased OMIC approaches [34]. Members of this study have led OMIC approaches that describe sets of molecular biomarkers associated with frailty status. To further characterize their link to frailty, we assessed their expression in a supervised, multicomponent, 16‐week exercise training programme.

In line with the growing evidence confirming that personalized exercise and multimodal interventions are beneficial and minimally invasive strategies to counteract the progressive physiological and functional decline of older adults [18], functional assessments revealed significant improvement across all scales, including SPPB, grip strength, gait speed and the frailty phenotype. Importantly, the improvement in physical activity among participants in the exercise group was accompanied by a reversal in the majority of biomarkers from both FRAILOMIC and BIOFRAIL studies. Among the FRAILOMIC biomarkers, this reversal was especially significant for *miR125b* and troponin T levels, which were two of the markers most consistently and significantly associated with frailty status according to the meta‐analysis of the four cohorts [12]. In BIOFRAIL, the restoration was particularly notable in the levels of NSF and in the core set of genes formed by EGR1, *DDX11L1* and *miR454*, which, as described in an additional manuscript, are associated with frailty and clinical adverse outcomes linked to frailty.

Clinical trials testing the efficacy of interventions are typically evaluated based on their overall or average effect, but often a substantial proportion of participants are either non‐responders or, in the worst case, are actively harmed by the intervention [35]. Thus, to explore this issue further, we performed a latent class analysis based on frailty characteristics, which identified two different clusters: responders and non‐responders. The relevance of each biomarker in the classification of these clusters was studied using the SVM model, which revealed that the three biomarkers contributing most strongly to the model and linked to the intervention response were *miR454*, *DDX11L1* and EGR1, the minimum trio identified in the BIOFRAIL study.

The reversal of MultiOMIC frailty signatures following supervised exercise suggests a profound biological recalibration. In an additional manuscript, we linked the expression of the trio of BIOFRAIL genes—*miR454*, *DDX11L1* and EGR1—to cellular senescence and senescence pathways. Furthermore, the significant decline in pro‐inflammatory markers such as CXCL8 and damage indicators like Troponin T reinforces the role of exercise in restoring systemic homeostasis and mitigating the ‘inflammaging’ characteristic of the frail state [9]. Therefore, the results of both studies allow us to hypothesize that senescence and inflammation may be relevant biological processes underlying the MultiOMIC signatures and necessary for frailty reversal after interventions with exercise programmes. Supporting this hypothesis, senescence is a process increasingly recognized as a key mechanism of aging and multiple aging‐related conditions [36, 37]. Several studies in preclinical models have shown that targeted elimination of senescent cells, genetically or pharmacologically, delays aging, attenuates age‐related diseases and conditions and improves physical function and healthspan [38, 39, 40, 41, 42, 43]. Furthermore, exercise programmes have been shown to reduce senescence biomarkers, including critical drivers/pathways and senescence associated secretory phenotype (SASP) members [24, 25].

From a clinical perspective, our findings represent a transition of frailty assessment from purely functional scales to a molecularly‐driven monitoring approach. The ability of the SVM and logistic regression models to distinguish ‘responders’ from ‘non‐responders’ underscores the potential for these biomarkers to guide personalized intervention strategies. Future applications could include the use of these MultiOMIC panels as surrogate endpoints in exercise programmes. However, the exercise group presented a higher proportion of women, becoming a limitation of the study. The extension of these analyses to a bigger, balanced cohort of women and men and other ethnicities would reinforce the strength of these results and allow the generalization to other populations. Additionally, intermediate measurements and a follow‐up after the cessation of the exercise intervention would be interesting data in order to further strengthen the translational relevance of the identified biomarkers.

Overall, our results demonstrated a restoration of a pattern of molecular frailty biomarkers derived from independent MultiOMIC approaches after a multicomponent exercise intervention in older adults. Our study provides the first evidence in humans that frailty biomarkers identified through unbiased approaches are significantly modulated by a structured exercise programme and can predict the response to exercise.

## Conflicts of Interest

The authors declare no conflicts of interest.

## Supporting information


**Data S1:** Supporting information.
